# Enhancing patient self-management

**DOI:** 10.7448/IAS.17.4.19496

**Published:** 2014-11-02

**Authors:** Alain Volny-Anne

**Affiliations:** Paris, France

## Abstract

In the context of HIV as a chronic disease, care being delivered with more constrained budgets and being more complex as many patients are ageing, the need for disease self-management is increasingly recognized. Like in other chronic diseases, HIV care and treatment guidelines increasingly promote the enhancement of health management by patients, within a framework of closer collaboration with their healthcare providers. Therefore, patients progressively become key resources on which health systems should count for efficiency and improvement. Is this a principle or is this reality? We are aware that to count in healthcare, resources must be allocated the necessary support. How does the required support translate into patients’ real lives? Do patient education programmes respond adequately to these needs? Are chronic care models really applicable in contexts where care is not yet sufficiently coordinated, especially with regards to people who are ageing with HIV and the multiple types of care they need? How far should a patient take responsibility for making best of healthcare resources? From the perspective of a person living and ageing with HIV, the role of patients in the management of their own health will be discussed.

